# Bivariate linkage analysis of cholesterol and triglyceride levels in the Framingham Heart Study

**DOI:** 10.1186/1471-2156-4-S1-S62

**Published:** 2003-12-31

**Authors:** Xuyang Zhang, Kai Wang

**Affiliations:** 1 Department of Speech Pathology and Audiology, The University of Iowa, Iowa City, USA; 2 Department of Biostatistics, Division of Statistical Genetics, The University of Iowa, Iowa City, Iowa, USA

## Abstract

We performed a bivariate analysis on cholesterol and triglyceride levels on data from the Framingham Heart Study using a new score statistic developed for the detection of potential pleiotropic, or cluster, genes. Univariate score statistics were also computed for each trait. At a significance level 0.001, linkage signals were found at markers GATA48B01 on chromosome 1, GATA21C12 on chromosome 8, and ATA55A11 on chromosome 16 using the bivariate analysis. At the same significance level, linkage signals were found at markers 036yb8 on chromosome 3 and GATA3F02 on chromosome 12 using the univariate analysis. A strong linkage signal was also found at marker GATA112F07 by both the bivariate analysis and the univariate analysis, a marker for which evidence for linkage had been reported previously in a related study.

## Background

Elevated triglyceride and cholesterol levels are two risk factors for cardiovascular diseases. These risk factors are often correlated with each other. In order to map the possible pleiotropic/clustered genes underlying the inheritance of these two traits, we performed a bivariate linkage analysis using a score statistic developed by Wang [1]. This score statistic is asymptotically equivalent to the likelihood ratio statistic and is straightforward to compute. We apply this statistic to data from Cohort 1 and Cohort 2 of the Framingham Heart Study.

## Methods

### Data

Participants in Cohort 1 had up to 16 reported cholesterol levels, and up to 3 reported triglyceride levels. For participants in Cohort 2, cholesterol and triglyceride levels were reported up to 5 times. These two cohorts together provided 22,040 measurements on the cholesterol level and 9,155 measurements on the triglyceride level (including all repeated measurements on all individuals). Individuals who lacked any measurements of cholesterol level or triglyceride level were excluded. A single linear regression of cholesterol on age was fit across different individuals and different measurements. The residuals from the regression fit were averaged for each individual. This average was used as the age-adjusted cholesterol level for that individual. The same method was used to obtain age-adjusted triglyceride level for each individual. Sib pairs from the same nuclear family or from different nuclear families that belonged to the same pedigree were regarded as biologically unrelated. For the case of univariate traits, there are reports showing that treating dependent sib pairs as independent ones does not increase the type I error rate of the test [2].

All sib pairs in all the pedigrees in Cohort 1 and Cohort 2 were generated, but not all of these sib pairs were used at the same time due to missing marker data. Genetic Analysis Workshop 13 (GAW13) provided identity-by-descent (IBD) sharing probabilities for some relative pairs (including sib pairs) at each of the scanned markers. The IBD sharing probabilities for a sib pair were available only for some markers. To simplify the programming, we excluded those markers at which there were less than 1000 sib pairs whose IBD sharing probabilities were available. Then, for each chromosome, we used only those sib pairs whose IBD sharing probabilities were available for all the remaining markers on that chromosome. See Table 1 for a summary of the number of markers excluded and the number of sib pairs used for each chromosome.

### Analysis

The bivariate score statistic is computed based on the observed phenotypic data on sib pairs. The phenotypic data of a sib pair can be denoted by a vector of four (adjusted) measurements – cholesterol levels on sib 1 and sib 2, and triglyceride levels on sib 1 and sib 2. Let **x_i _** be the phenotypic data on the *i*^th ^sib pair and **Σ**_0_ be the sample variance-covariance of **x_i_**. As an average of the residuals of a regression, the sample mean of cholesterol levels on sib 1 and sib 2 is 0, so is the sample mean of triglyceride level. Let **Σ**_0_ be a 4 × 4 symmetric matrix whose (*i*,*j*) element is denoted by aij. Note that a_11 _ and a_33_ are the variances of the cholesterol and triglyceride levels, respectively, of the first sib in the pairs. Similarly, a_22_ and a_44_ are the variances of the cholesterol and triglyceride levels of the second sib in the sib pairs. The off-diagonal terms represent covariances: a_13 _= a_31_ is the covariance between cholesterol and triglycerides for the first sib in the sib pairs, and a_24_ = a_42_ is the covariance for the second sib in the sib pairs. Since the sib-sib relationship in a sib pair is symmetric, we expect that a_11_ ≈ a_22_, a_33_ ≈ a_44_ and a_13_ ≈ a_24_ when the sample size is large. Alternatively, we can also use the (adjusted) measurements on cholesterol and triglycerides on all sibs (do not distinguish sib 1 from sib 2) in calculating the entries of **Σ**_0_. Then there would be a_11_ = a_22_, a_33_ = a_44_, and a_13_ = a_24_. Since the sample size is fairly large, we expect both methods give similar **Σ**_0_.

Define

**w_i _**= (*w*_i1_, *w*_i2_, *w*_i3_, *w*_i4_)^t ^= **∑_0_^-1 ^x_i_**

and

*z*_i _= *w*_i1_*w*_i2_*a*_11 _+ (*w*_i1_*w*_i3 _+ *w*_i2_*w*_i4_)*a*_13 _+ *w*_i3_*w*_i4_*a*_33_.

Denote the proportion of alleles that are shared IBD by the *i*^th ^sib pair by π_i_. Let  and  be the sample means of {π_i_} and {z_i_}, respectively. Define



where *N *is the total number of sib pairs. When the putative locus is not linked to any trait locus, the expectation of *b *is 0 and its variance is Var(b) = N s^2^_π_s^2^_z_, where s^2^_π _and s^2^_z _are the sample variances of {π_i_} and {z_i_}, respectively. The score statistic S for the bivariate phenotypes is defined by *S *= *b*^2^/*Var*(*b*) if *b *> 0; *S *= 0 otherwise. When the putative locus is not linked to any quantitative trait loci (QTL), the asymptotic distribution of this one-sided tests statistic, *S*, is 0.5 χ^2^_0 _+ 0.5 χ^2^_1 _[1]. The score statistic *S *is a special case described by Wang [1] – the locus specific variances and covariance for the two traits are assumed to be proportional to their total variances and covariance.

## Results

**Figure 1:**
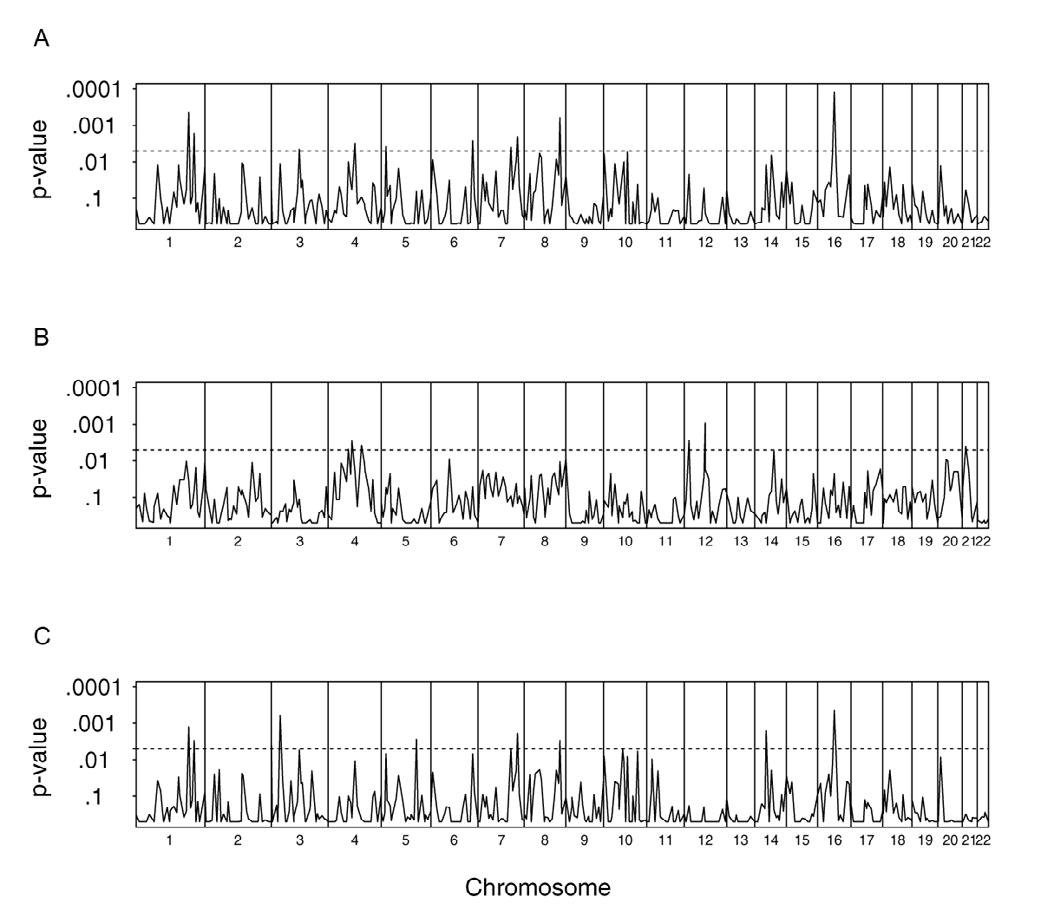
***p*-values for the three statistics throughout the genome **Bivariate score statistic (A), univariate score statistic for adjusted cholesterol level (B), and univariate score statistic for adjusted triglyceride level (C).

The score statistic *S *was calculated for every screened marker. In addition, the univariate score statistic of Wang and Huang [3] was also calculated for cholesterol level and triglyceride level separately. For sib-pair data, the type of data used in our analyses, this univariate score statistic is equivalent to other methods [4,5]. The *p*-values of these three score statistics (one for the bivariate phenotypes, one for each of the two univariate phenotypes) at each marker location are plotted in Figure 1. Markers with *p*-values less than the significance level of α = 0.005 are shown in Table 2.

At the significance level 0.005, 10 markers were identified by the bivariate score statistic: 2 each from chromosome 1 (at 212 cM and 233 cM) and 7 (at 128 cM and 155 cM), and 1 each from chromosome 3 (at 112 cM), 4 (at 105 cM), 5 (at 19 cM), 6 (at 166 cM), 8 (at 140 cM), and 16 (at 64 cM). Five out of the 10 markers were also identified by the univariate score statistic for the adjusted triglyceride level. They were the two on chromosome 1, one on chromosome 7 (at 155 cM), one on chromosome 8, and one on chromosome 16. None of the 10 markers were identified by the univariate score statistic for the age-adjusted cholesterol level. The results seem to suggest that there were large overlaps of linkage signals between the bivariate score statistic and the univariate score statistic for the age-adjusted triglyceride level. There were no overlaps of linkage signals between the bivariate score statistic and the univariate score statistic for the age-adjusted cholesterol level. There were 5 markers that were identified by the bivariate score statistic, but not identified by any of the univariate score statistics. There were 3 markers whose *p*-values were below 0.001: one on chromosome 1 at 212 cM, one on chromosome 8 at 140 cM, and the other on chromosome 16 at 64 cM. The regions suggested by these 3 markers may be investigated in future genotyping and analysis.

## Discussion

We performed a bivariate analysis of cholesterol and triglyceride levels on sib-pair data from the Framingham Heart Study using a method recently developed by Wang [1]. This method is asymptotically equivalent to the likelihood ratio statistic, but is straightforward to calculate. We also calculated the univariate score statistics for cholesterol and triglyceride levels separately. Five markers were identified by both the bivariate score statistic and the univariate score statistic for the adjusted triglyceride level, while the results of the bivariate score statistics had no overlap with the univariate score statistic for the age adjusted cholesterol levels.

The method in Wang [1] is general enough to handle general pedigrees, but we only applied it to sib pairs that were extracted from general pedigrees. This is because the programming for sib pairs is relatively easy and was feasible given the time constraint for GAW13. Some linkage information may have lost due to the fact that dependent sib pairs were treated as independent sib pairs, but the type I error rate of the test statistic is expected to be valid.

In a related study, Shearman et al. [6] used the ratio of triglyceride level to high-density lipoprotein cholesterol level as the phenotype of interest. Linkage evidence was reported at marker GATA112F07 (155 cM on chromosome 7), a marker that resulted in a *p*-value 0.0020 for the bivariate score statistic used in the current report. These authors reported a LOD score 1.5 at 70 cM on chromosome 16 with multipoint mapping. We used single-point IBD sharing probabilities with the bivariate score statistic and obtained a significant linkage signal (*p *= 0.0001) for marker ATA55A11 (64 cM on chromosome 16), 6 cM away from the locus they identified. Other markers in Table 2 that have small *p*-values for the bivariate or univariate score statistics but that did not show evidence for linkage in Shearman et al. [6] include GATA48B01, 036yb8, GATA21C12, and GATA3F02.

One caveat about bivariate analyses is that they are not always more powerful than univariate analyses. Theoretical [7] and simulation studies [1,8,9] demonstrate that when the polygenic correlation is in the same direction as the major gene correlation, a bivariate analysis may have lower power than a univariate analysis.

## Figures and Tables

**Table 1:** Number of markers available and analyzed on each chromosome

**Chr**	**Markers Provided**	**Markers Analyzed**	**Sib Pairs^A^**
1	32	30	965
2	30	28	981
3	27	27	380
4	21	21	1215
5	26	24	719
6	23	19	1187
7	22	20	1245
8	19	19	1298
9	19	17	401
10	22	20	970
11	17	15	788
12	19	18	1178
13	12	11	1524
14	17	14	1292
15	13	12	1318
16	14	14	1218
17	16	15	1284
18	15	14	1291
19	11	10	1426
20	11	10	1515
21	6	6	1798
22	7	6	1843

^A^The sample size is the same for all markers on the same chromosome.

**Table 2:** Summary of markers that are significant at significance level 0.005

Chr.	Marker	p-telomere (cM)	Bivariate Score statistic	Univariate score statistic	
				
				Cholesterol	Triglyceride
1	Gata48B01	212	11.029(.0004)		9.141(.0013)
1	GATA87F04	233	8.618(.0017)		7.602(.0029)
3	3PTEL25	1			8.358(.0019)
3	036yb8	37			10.445(.0006)
3	GATA128C02	112	6.799(.0046)		
4	GATA24H01	78		6.670(.0049)	
4	ATA2A03	93		7.665(.0028)	
4	GATA2F11	105	7.534(.0030)		
4	ATA26B08	130		7.159(.0037)	
5	GATA2A04	19	7.119(.0038)		
5	GATA2H09	139			7.682(.0028)
6	242zg5	166	7.776(.0026)		
7	GGAA6D03	128	7.036(.0040)		
7	GATA112F07	155	8.274(.0020)		8.410(.0019)
8	GATA21C12	140	10.474(.0006)		7.603(.0029)
12	GATA49D12	18		7.695(.0028)	
12	GATA3F02	81		9.674(.0009)	
14	GATA4B04	44			8.648(.0016)
16	ATA55A11	64	13.489(.0001)		11.049(.0004)
21	GGAA3C07	13		7.043(.0040)	
